# From Microstates to Macrostates in the Conformational Dynamics of GroEL: A Single-Molecule Förster Resonance Energy Transfer Study

**DOI:** 10.1021/acs.jpclett.3c01281

**Published:** 2023-07-13

**Authors:** Demian G. Liebermann, Jakub Jungwirth, Inbal Riven, Yoav Barak, Dorit Levy, Amnon Horovitz, Gilad Haran

**Affiliations:** ^†^Departments of Chemical and Biological Physics, ^‡^Chemical and Structural Biology, and ^§^Chemical Research Support, Weizmann Institute of Science, Rehovot, 76100, Israel

## Abstract

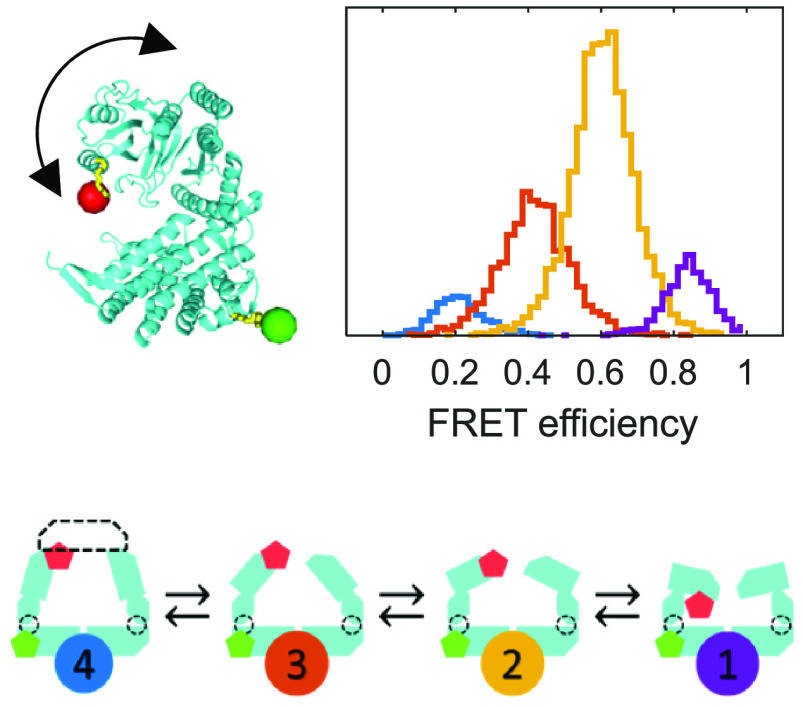

The chaperonin GroEL is a multisubunit molecular machine that assists in protein folding in the *Escherichia coli* cytosol. Past studies have shown that GroEL undergoes large allosteric conformational changes during its reaction cycle. Here, we report single-molecule Förster resonance energy transfer measurements that directly probe the conformational transitions of one subunit within GroEL and its single-ring variant under equilibrium conditions. We find that four microstates span the conformational manifold of the protein and interconvert on the submillisecond time scale. A unique set of relative populations of these microstates, termed a macrostate, is obtained by varying solution conditions, e.g., adding different nucleotides or the cochaperone GroES. Strikingly, ATP titration studies demonstrate that the partition between the apo and ATP-ligated conformational macrostates traces a sigmoidal response with a Hill coefficient similar to that obtained in bulk experiments of ATP hydrolysis. These coinciding results from bulk measurements for an entire ring and single-molecule measurements for a single subunit provide new evidence for the concerted allosteric transition of all seven subunits.

Molecular machines participate in multiple biological functions in the cell, such as the synthesis of biomolecules, cargo transportation, and DNA replication, to name a few. Such actions often involve converting available chemical energy, e.g., in the form of ATP, into conformational changes with allosteric fine-tuning mechanisms.^1^ A prominent case study for investigating allosteric molecular machines is the oligomeric chaperonin GroEL, which assists in protein folding in the *Escherichia coli* cytosol in an ATP-dependent fashion.^2−4^

First identified as a heat-shock protein,^5,6^ GroEL is essential for the successful folding of certain proteins in the cell under either normal or stress conditions.^7−10^ It consists of 14 identical subunits arranged in two stacked heptameric rings, each forming a folding cavity that can accommodate a non-native protein substrate.^11−14^ The GroEL subunit can be segmented into three functional domains (Figure 1A). The equatorial domain contains the nucleotide binding pocket^15,16^ and forms the inter-ring interface.^13,17^ The apical domain is involved in the interaction with non-native protein substrates^18−20^ and the cochaperone GroES, a heptameric complex that occludes the folding cage with the protein substrate inside.^12^ Finally, the intermediate domain connects the equatorial and apical domains and plays an essential role in the activity of the protein.^16^

**Figure 1:**
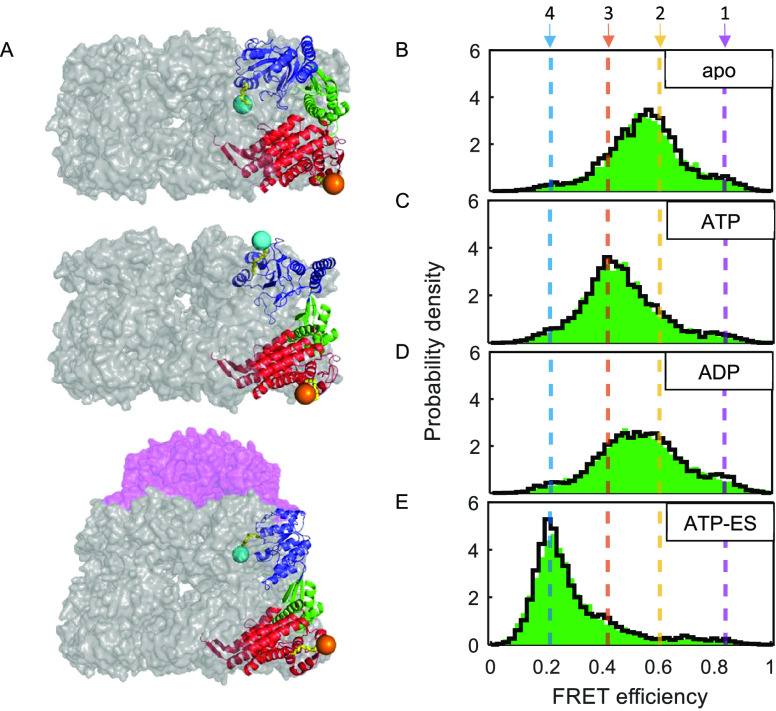
(A) Structural models of the GroEL subunit within a single ring in different conformational states. From top to bottom: apo GroEL (PDB: 1XCK),^33^ GroEL bound to ATP (PDB: 1AAR),^35^ and GroEL bound to GroES (PDB: 1AON).^12^ Shown are the equatorial (red), intermediate (green), and apical (blue) domains of one GroEL subunit. GroES is shown in magenta. In each structure, a particular risFRET realization of the fluorescent labels is shown, with the linkers in yellow, and cyan and orange spheres as the dye moieties at positions E255 and D428, respectively. The interdye distance and expected FRET efficiency values from the risFRET simulations are *R* = 56 Å, *E* = 0.45 for the apo model, *R* = 67 Å, *E* = 0.25 for the ATP-bound model, and *R* = 87 Å, *E* = 0.065 for the GroES-bound model. (**B–E**) FRET efficiency histograms of SR1 E255C/D428C (green) under the following conditions: apo (B), 1 mM ATP (C), 1 mM ADP (D), and 1 mM ATP + GroES (E). The overlaid solid black lines are the recolored histograms generated using the model parameters from the global H^2^MM analysis (see text). The arrows and dashed lines mark the FRET efficiency values of the four microstates obtained from the analysis, which are 0.850 ± 0.002, 0.608 ± 0.005, 0.419 ± 0.006, and 0.213 ± 0.002 (mean ± standard error), respectively.

The complete reaction cycle of GroEL is governed by a seconds-long time window set by the hydrolysis of ATP and the consequent release of ADP/P_i_,^21^ during which an encapsulated non-native protein substrate can start folding into its native state via one of several putative mechanisms.^22−29^ The allosteric transitions of GroEL with respect to ATP binding have been described by a nested cooperativity model, which assigns positive cooperativity between the seven subunits of one heptameric ring and negative cooperativity between the two stacked heptameric rings.^30,31^ The intraring concerted transitions are described by the Monod–Wyman–Changeux (MWC) formalism,^32^ which posits an equilibrium between two allosteric states, T (tense) and R (relaxed), with low and high affinity toward ATP binding, respectively.

X-ray crystallography^11,12,14,33,34^ and cryo-EM^13,35−37^ structures reveal that GroEL samples a rich conformational space with large domain motions during its reaction cycle. Upon ATP binding, the GroEL subunits transition from a closed to an open conformation with an upward motion of their apical domains. Further upward and clockwise twisting of the apical domains occurs when GroEL binds to the GroES heptamer. While most available structures were solved with an imposed C7 symmetry, in line with the concerted allosteric transition from the T to R allosteric states, a few studies have also shown stable asymmetric GroEL configurations, suggesting conformational heterogeneity among the protein subunits.^34,36^

Kinetic investigations of GroEL have identified conformational processes spanning several orders of magnitude in time. Relatively slow events, such as substrate binding and release^38^ or GroES exchange^16,21^ coupled to ATP hydrolysis, have been shown to occur within a few seconds. Other bulk transient kinetic experiments have indirectly probed conformational changes in the 10–200 ms range,^39−42^ showing that the allosteric transitions triggered by ATP binding involve multiple kinetic phases.

Despite multiple studies on the allosteric transitions of GroEL, a detailed, time-resolved description of domain motions within its subunits has not been presented to date, mainly due to the difficulties in collecting direct, highly resolved spatial and temporal data and the limitations of ensemble measurements. In this work, we address this deficiency and study the conformational dynamics of the GroEL subunit by measuring the local *equilibrium molecular motions* of a single subunit within the heptamer, using single-molecule Förster resonance energy transfer (smFRET) experiments on a single-ring variant of GroEL.^43^ Analyzing the data with a photon-by-photon statistical algorithm based on hidden Markov models,^44^ we find that the GroEL subunit transitions between four conformational microstates on a submillisecond time scale and that the relative populations of these states depends on external conditions such as the presence and identity of nucleotides or GroES. Analysis of an ATP titration curve generated from the single-molecule data sets provides direct evidence for the interrelation between the conformational states of the GroEL subunit and the allosteric transitions of the machine during its functional cycle.

In order to study the dynamics of a GroEL subunit using smFRET spectroscopy, we first searched for positions on the protein that, when labeled, would well sample its conformational changes. To that end, we used a program developed in the lab, risFRET (see Searching for a Labeling Site Using risFRET in the Methods section in the Supporting Information), which builds models of the fluorescent probes onto PDB structural models of a protein and outputs a ranked list of potential FRET pairs. The ranking considers the expected FRET efficiency change between two specified conformations, the surface accessibility of the probes, and the conservation of residues.^45^ We eventually selected two labeling sites in GroEL based on this analysis, E255 in the apical domain and D428 in the equatorial domain. Figure 1A shows three structural models of the GroEL heptameric ring with a labeled subunit in the apo, ATP-bound, and GroES-bound states, each with an illustrative risFRET realization for the fluorescent dyes AlexaFluor 488 and AlexaFluor 594, shown as spheres attached to the labeling positions with their linkers.

Throughout this work, we mainly used the single-ring variant of GroEL, SR1,^43^ which, like wild-type (WT) GroEL, exhibits ATPase activity with positive cooperativity, as shown in Figure S2A. SR1 also interacts with the cochaperone GroES in the presence of ATP. However, due to a lack of a negative allosteric signal from an opposite GroEL ring,^16^ the SR1-GroES complex remains stable for more than a hundred minutes.^13,24,43,46^ This property was utilized in our experiments. To detect FRET signals originating from only one GroEL subunit in the heptameric ring complex, we prepared SR1 constructs with a single double-labeled subunit using a disassembly reassembly procedure (see Purification of GroEL Variants, Protein Labeling, and Reassembly Procedure in the Methods and Figure S1). Briefly, purified and labeled E255C/D428C monomers were mixed with unmodified SR1 subunits in a stoichiometric ratio of 1:100 in the presence of 4 M urea. After a short incubation, the mixture of monomers was dialyzed in a reassembly buffer, allowing the formation of SR1 rings. This mixing ratio guaranteed that ∼97% of the labeled SR1 complexes contained a single subunit labeled with the fluorescent dyes.

We performed a series of control experiments on the labeled and unlabeled SR1 constructs. An SR1 construct with seven unlabeled E255C/D428C subunits demonstrated essentially the same ATPase activity as the WT SR1 variant, with a rate constant of 43 ± 2 min^–1^ and a cooperative response curve with a Hill coefficient of 2.0 ± 0.2 (Figure S2A-B, see Steady-State Kinetic Assay in Methods). The integrity of various SR1 heptameric constructs used in this work was confirmed by comparing their migration pattern on a native gel and by native mass spectrometry measurements (Figure S2C–E, see Denaturing and Native Gel Electrophoresis and Native Mass Spectrometry in Methods). The rotational freedom of the donor and acceptor dyes at each labeling site was verified with fluorescence anisotropy measurements on SR1 constructs with a single labeled protomer (see Fluorescence Anisotropy in Methods). Steady-state fluorescence anisotropy measurements of the labeled SR1 construct yielded values of 0.17–0.27 that did not change in the presence of ATP, ADP, and GroES (Table S1). Inspection of time-resolved anisotropy decay curves of each construct in the apo state showed a fast decay process with a correlation time of ∼1 ns (Figure S3), attributed to the rotation of the dyes, and a slow correlation time attributed to the rotation of the protein itself.^47^ These results indicate that the fluorescent labels are free to rotate on the protein, suggesting that changes in FRET efficiency observed in the experiments are only due to distance changes.^48^ We also validated reported observations^49−51^ that the three native cysteine residues of SR1 have a low labeling efficiency by attempting to label and prepare the WT variant with the same procedures as the single and double-labeled SR1 variants and conducting smFRET experiments (see Labeling Controls on Native Cysteine in Methods). Single-molecule stoichiometry histograms (Figure S2F) demonstrated that the insertion of surface-exposed cysteine residues into SR1 reduced the labeling of the native cysteines significantly. Based on these results, we found the labeled SR1 construct to be a suitable model for studying the allosteric conformational changes of a single GroEL subunit within the heptameric ring.

We conducted pulsed-interleaved excitation smFRET experiments on diluted samples containing the labeled SR1 construct without substrates (apo) or in the presence of either 1 mM ATP, 1 mM ADP, or 1 mM ATP with a stoichiometric excess of GroES relative to GroEL (see Figure S4 and Single-Molecule Experiments in Methods). In these measurements, labeled SR1 complexes freely diffused through a focused excitation laser beam, one molecule at a time, and emitted bursts of photons, also termed photon trajectories (see below). The arrival times and the colors of the detected photons in each burst were recorded, and the FRET efficiency and stoichiometry were calculated. Under each condition, 2–3 measurements were conducted. Figure 1B–E shows FRET efficiency histograms (green) of selected measurement repeats (see Figure S5 for corrected FRET efficiency histograms and Figure S6 for uncorrected FRET efficiency histograms from all measurement repeats). The rich conformational space of the GroEL subunit detected in all smFRET experiments is evident from the broad histograms, suggesting several distinct substrate-dependent conformational states. In the apo state (Figure 1B), the FRET efficiency distribution exhibits a broad major peak centered at ∼0.55. In the presence of excess ATP (Figure 1C), the FRET efficiency distribution is shifted to lower values with the major peak now centered at ∼0.4, indicating that the apical domain is extended to a more open conformation. Interestingly, the addition of excess ADP (Figure 1D) resulted in a broader FRET efficiency distribution centered between the apo and ATP peaks at ∼0.5. A dramatic shift in the FRET efficiency histogram was observed when the cochaperone GroES was added in addition to excess ATP (Figure 1E), with the peak value of the histogram now found at ∼0.2.

Since ATP bound to SR1 can hydrolyze to ADP, which may be released only slowly from the complex, it was possible that the recorded FRET efficiency distributions in the presence of ATP and ATP-GroES reflected a mixture of subunits bound to ATP and ADP. To examine this possibility, we conducted the same smFRET experiments in the presence of the ATP analog ADP-beryllium-fluoride^52^ (ADP-BeF_x_, where x is the number of F atoms; see Preparation of GroEL with ATP-Analog in Methods). GroEL bound with this analog has been shown to mimic ATP-bound GroEL, with the BeF_x_ moiety acting as a stable substitution for the γ-phosphate of ATP.^38,53−55^ The FRET efficiency histograms in the presence of ADP-BeF_x_, shown in Figure S7, are similar to those of the ATP-bound chaperone, indicating that ATP hydrolysis does not lead to the appearance of additional populations. Instead, these results confirm that the GroEL subunit can adopt several conformations even when it is indefinitely bound to ATP and GroES. In addition, these observations further emphasize the role of the γ-phosphate moiety in stabilizing the open, extended GroEL conformations. ADP alone does not seem to induce open conformations as effectively as ATP or ATP+GroES.

To verify that the observed conformational behavior of the SR1 heptameric ring reflects the behavior of the double-ring GroEL complex, we conducted smFRET experiments on the double-ring construct with the same labeling positions (prepared using the same reassembly procedure). The FRET efficiency histograms shown in Figure S5A–D indicate that the two GroEL variants populate similar FRET efficiency states but with different amplitudes, as seen predominantly in the case of double-ring GroEL in the presence of excess ATP and GroES. These differences can be explained considering that in the smFRET experiment of the double-ring variant, half of the detected labeled subunits are in a GroES-bound (*cis)* ring, while the other half are in an unbound (*trans)* ring. This assertion was validated by forcing the double-ring variant to adopt the so-called symmetric football complex,^55^ where the two GroEL rings are bound to GroES (Figure S5E). Since all 14 subunits now adopted the same GroES-bound state in this construct, we obtained a histogram that is more similar to the case of GroES-bound SR1. These observations strongly indicate that the conformational space sampled by a subunit in the heptameric ring is similar to that sampled by a subunit in the double-ring GroEL complex.

The labeled SR1 construct thus exhibits conformational behavior that matches expectations based on prior knowledge. However, the existence of broad FRET efficiency distributions indicates that, during the different phases of its reaction cycle, the GroEL subunit transitions between several conformational states, with dynamics on the time scale of ∼0.5–1 ms, which is the typical passage time of a molecule diffusing through the laser beam in the smFRET experiment.

To obtain a detailed description of the underlying conformational dynamics of the GroEL subunit, we performed a photon-by-photon hidden Markov model analysis of single-molecule photon trajectories using the program H^2^MM^44^ (see H^2^MM Analysis in Methods). For each data set, which consists of ∼6000–10000 photon trajectories, the H^2^MM analysis uses a kinetic model with a discrete number of conformational states and finds the FRET efficiency value and population of each state, as well as the state-to-state transition rates that best describe the data. We found that a four-state Markov chain model allowed us to globally account for all experimental data sets (Figure S8, see H^2^MM Analysis in Methods), with the FRET efficiency values shared among all data sets. This analysis suggested that SR1 samples the same four conformations, which we term ***microstates***, under all conditions.

We used several methods to validate the results of the optimization. First, we performed photon recoloring simulations, in which simulated photon trajectories are generated based on the H^2^MM parameters and the photon arrival times from measured data sets^56^ (see H^2^MM Analysis in Methods). The simulated data were then used to generate recolored FRET efficiency histograms, which are shown in Figures 1B–E as solid black lines on top of the raw data histograms (green) (see Figure S6 for recolored histograms of all repeats and conditions). The good overlaps between the experimental and recolored histograms demonstrate that the model parameters found in the H^2^MM analysis describe the smFRET experimental data well. Second, we validated the models using dwell-time analysis.^57^ In this method, likelihood-weighted state-to-state dwell-time distributions are generated based on the data and the H^2^MM parameters (see Weighted Dwell-Time Analysis in Methods), and the mean dwell-time values for the four states are extracted by fitting these distributions to monoexponential functions. The very good agreement between the dwell-time values obtained from this analysis (Figure S9) and those calculated directly from the H^2^MM parameters, which can be observed in Table S2, provides additional validation of the analysis results. Further, we performed segmentation analysis using the Viterbi algorithm, which generates a realization of the most likely state sequence for each photon trajectory based on the H^2^MM parameters (see Burst Segmentation in Methods). We selected only photon trajectories that were assigned to a single microstate and plotted their histograms (Figure S10). The good separation of the microstate histograms validates the H^2^MM analysis state assignmnet. Finally, the presence of submillisecond conformational dynamics was confirmed in a burst-wise fluorescence correlation analysis (see Burst-Wise Fluorescence Correlation Analysis in Methods), showing a characteristic increase of the correlation curves at time scales matching the H^2^MM values (Figure S11).

The FRET efficiency values of the four states obtained from the H^2^MM analysis are 0.850 ± 0.002, 0.608 ± 0.005, 0.419 ± 0.006, and 0.213 ± 0.002, and they are marked as dashed colored lines in Figures 1B–E (FRET values corrected for donor leak and direct excitation of acceptor are ∼0.83, ∼0.55, ∼0.33, and ∼0.09, see H^2^MM analysis in Methods for error estimation). The four ***microstates*** are labeled numerically according to decreasing order of their FRET efficiency values. The measured and corrected FRET efficiency values of the four microstates can be qualitatively compared to published conformations of GroEL.^12,33,35^ In particular, it is found that microstate 2 matches the structural model of GroEL under the apo condition (Figure 1A, upper), while microstate 3 matches the nucleotide-bound conformation (Figure 1A, middle) and microstate 4 matches the GroES-bound conformation (Figure 1A, bottom). We did not find an existing structural model that matched microstate 1, probably because it is relatively infrequently populated. The state-to-state transition rates obtained from the H^2^MM analysis are shown in a kinetic scheme in Figure 2A–D. Under all measured conditions, these transition rates correspond to conformational dynamics on the millisecond to submillisecond time scales, 3 orders of magnitude faster than the bulk ATP hydrolysis rate of SR1 (compare with Figure S2A,B). These fast transition rates provide an explanation for the emergence of broad FRET efficiency histograms.

**Figure 2:**
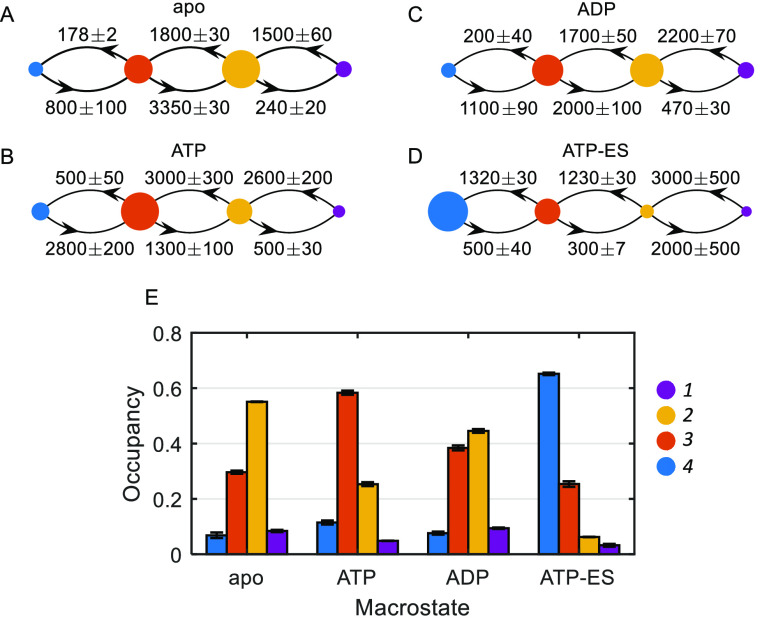
(A–D) Markov chain model graphs of various GroEL macrostates. The four microstates are represented with color-coded nodes. The area of each node is scaled to the mean fractional occupancy of each microstate. Transition rates in units of s^–1^ are shown above/below the arrows. (E) Microstate fractional steady-state populations for each of the macrostates, calculated from the transition-rate matrix. See Table S3 for numerical values. Errors represent standard error of the mean derived from repeats of the experiment. Microstates are represented with colors as specified.

The steady-state population values for each of the four microstates, directly obtained by diagonalizing the transition rates matrices, are shown in Figure 2E and Table S3. The distribution of microstate probabilities under each condition gives rise to a conformational fingerprint that we term a ***macrostate***. In the apo macrostate, the GroEL subunit spends 55.1 ± 0.1% and 29.6 ± 0.6% of the time in microstates 2 and 3, respectively, while microstates 1 (8.4 ± 0.5%) and 4 (7.0 ± 1.0%) are visited less frequently. A notable population shift is seen in the ATP macrostate, resulting in a decrease in the population of microstate 2 to 25.3 ± 0.7% and an increase in that of microstate 3 to 58.4 ± 0.7%. Notice also the slight occupancy increase for microstate 4 (11.5 ± 0.7%). In the ADP macrostate, the observed broad FRET efficiency distribution seen in Figure 1D can be traced to the similar occupancies of microstates 2 (44.6 ± 0.7%) and 3 (38.4 ± 0.9%). In the ATP-ES macrostate, the GroEL subunit resides primarily in microstate 4 (65.2 ± 0.4%) but also in the higher FRET efficiency microstates, with microstate 3 populated 25.4 ± 1.0% of the time.

We investigated the relationship between the observed subunit conformational dynamics and the allosteric transitions of GroEL with respect to ATP by conducting a series of smFRET experiments on samples with increasing ATP concentrations (see smFRET ATP Titration and Burst–Wise–Likelihood Analysis in Methods). Since depletion of ATP was expected in experiments with low nucleotide concentrations, the samples contained an enzymatic ATP regeneration system, which ensured that the steady-state conditions were maintained throughout the measurements. Figure 3 A shows FRET efficiency histograms of SR1 at various ATP concentrations, segmented into the four microstates as discussed above (see also Burst Segmentation in Methods), demonstrating the gradual changes in their populations. To trace the allosteric response, we used a likelihood test to assign each measured molecule to either the apo or ATP macrostate. This procedure was performed for all data sets in this series, and the mean likelihood scores for the apo and ATP macrostates were calculated. Using the scaled score of the ATP macrostate, we generated an ATP saturation curve, as shown in Figure 3B. Remarkably, the plot reproduces the sigmoidal curve familiar from the bulk ATP hydrolysis titration experiment of SR1 (compare with Figure S2B). A fit to a Hill function provides essentially the same Hill coefficient (2.9 ± 0.1) as that obtained in the bulk ATP hydrolysis experiment (2.8 ± 0.1).

**Figure 3:**
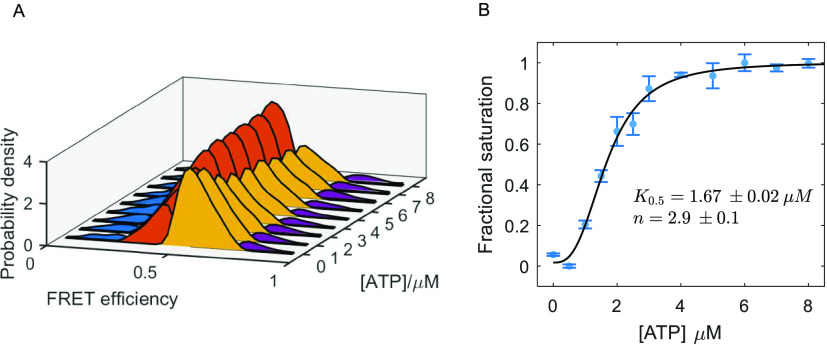
(A) Segmented histograms of SR1 at different ATP concentrations. The distribution of each microstate is shown with the same color code used in previous figures. Histograms were smoothed using a Gaussian filter with a window size of 5 for visualization purposes. (B) Fractional saturation vs ATP concertation calculated using the likelihood score. The experimental points (blue) were fitted to the Hill equation (black curve), and the resulting parameters are shown in the inset.

The experimental results reported above describe the direct monitoring of the conformational dynamics of a functioning GroEL molecule. In particular, we used time-resolved smFRET spectroscopy to observe the fluctuations of a single subunit. Photon-by-photon H^2^MM analysis of the experimental data yields a dynamic model for the conformational transitions. Surprisingly, the GroEL subunit is found to undergo domain motions on the microsecond-millisecond time scale, significantly faster than the functional cycle of the chaperone. These conformational transitions involve four microstates, whose populations under each of the probed equilibrium conditions give rise to a dynamic pattern that we term a macrostate. Our modeling approach thus provides a way to characterize the operational phases of GroEL and its allosteric behavior in terms of the subunit conformational dynamics. Past experiments have probed the kinetics of the GroEL cycle in the bulk.^38−41,51^ However, while the conformational dynamics of GroEL have been studied in computer simulations,^58−62^ a direct, time-resolved measurement of domain motions of the chaperonin has so far been missing.

Our findings clearly demonstrate that the GroEL subunit adopts multiple conformations under all measured conditions. The characteristic microstate population partition in each macrostate sheds light on the relation between the ensemble of conformations sampled by the GroEL subunit and the functional tasks that the machine performs during its reaction cycle. In the nonligated apo macrostate, the GroEL subunit transitions between the two most populated microstates, 2 and 3, on a time scale of ∼300–500 μs (Figure 2 A and Table S2). According to the steady-state partition in the apo macrostate (Figure 2E and Table S3), the GroEL subunit resides 55% of the time in microstate 2, which, as already mentioned, resembles the subunit conformation seen in the nonligated structure (Figure 1A). In this closed conformation, the hydrophobic helices of the apical domains face toward the cavity,^33^ collectively forming a hydrophobic binding surface to which the misfolded protein substrate can bind with a higher affinity.^18,20^ By dynamically sampling the closed microstate 2 as well as microstate 1, which is likely even more compact, the GroEL subunit is ready for a substrate binding event. Nevertheless, it still retains the possibility of converting to the more open conformational microstates (3 and 4), which could be essential for subsequent steps such as ATP hydrolysis or GroES binding.^63^

Considering the millimolar physiological concentrations of ATP or ADP in the *E. coli* cytosol,^64^ we can expect the GroEL machine to be mostly bound to these nucleotides, which implies that the machine resides in the ATP and ADP macrostates, respectively. The GroEL subunit continues to exhibit dynamic behavior in the two nucleotide-bound macrostates, with transitions at ∼300–500 μs (Figure 2B,C and Table S2). Domain motions with similar time scales were reported in simulations probing ligand-induced conformational changes from the apo to the ATP-bound states.^60^ In the ATP macrostate, as opposed to the apo macrostate, the subunit resides mostly in the microstates with the lower FRET efficiency values (3 and 4), as expected based on structural information (Figure 1A).^34,35,65^ In such conformations, the apical domain is now extended upward, and the continuous binding surface, prominent in the apo structure, is disrupted. These conformational states diminish the avidity toward the protein substrate,^18^ which in turn promotes its subsequent release into the GroEL cavity or back to the solution. Also note that the presence of ATP increases the population of the fully extended, open microstate 4 from ∼7% to ∼11%, which, in line with the conformational selection model,^66^ could enhance the likelihood of fruitful interaction between the apical domains of GroEL and the loops of the cochaperone GroES in subsequent steps of the cycle.^12^ In the ADP macrostate, which ideally emulates the stage after ATP hydrolysis and the release of phosphate and GroES, the subunit shows similar occupancies for the closed (1 and 2) and open (3 and 4) conformational microstates. Considering the ADP-bound *trans*-ring in the asymmetric reaction cycle of double-ring GroEL,^37,67^ such a partition could be advantageous, as it allows the GroEL subunit to readily switch to the closed conformational microstates, so that it can reform the high-affinity binding surface,^20^ thereby priming the GroEL ring for a subsequent binding event of a non-native protein substrate.

The behavior of the GroEL subunit in the ATP-ES macrostate is, surprisingly, not static, and pronounced conformational transitions were detected in our experiments, even though the subunit is supposedly “locked” in the long-lived SR1-GroES complex (Figure S7).^13,24,43,46^ In fact, while the open microstate 4 was predominantly populated in this macrostate, conformational transitions to the more closed microstates were also detected (Table S2). A possible explanation for the persistent dynamics in the ATP-ES macrostate is that the apical domain of the subunit occasionally detaches from the GroES loops and is thus free to transition to all other microstates, while the other subunits maintain GroES in place by their collective contributions to avidity. Another possibility is that the equatorial domains are more flexible in SR1.

The ATP titration curve, generated based on the likelihood score analysis of individual molecules and using the apo and ATP macrostate models, traces the familiar sigmoidal shape for positive cooperativity (Figure 3B). This result directly demonstrates that the conformational motions sampled in the single-molecule experiment of an individual GroEL subunit are associated with the allosteric mechanism of the entire complex. While the functional cycle of GroEL takes place out-of-equilibrium, as ATP is continuously hydrolyzed, it is expected that fluctuations in equilibrium will sample the same states on the energy landscape sampled in the cycle. Moreover, the Hill coefficient value obtained in this experiment (2.9 ± 0.1) matches well, within error, the value obtained from the bulk ATP titration experiment of SR1 WT (2.8 ± 0.1) (Figure S2A). This striking agreement of the Hill coefficients from two widely different observables—the bulk steady-state ATP hydrolysis rate of the SR1 complex and the local conformational changes of a single labeled subunit—is strong evidence that the allosteric transition in GroEL involves transitions between two states without intermediates, matching the concerted model. Such a similarity in Hill coefficient values from different observables was also reported in ensemble kinetic studies on double-ring^39−41^ and single-ring^42,46^ GroEL. Another interesting consequence of locally measuring a single GroEL subunit is manifested by the apparent binding constants obtained from fits to the Hill function. In the smFRET ATP titration experiment, the apparent binding constant is ∼1.7 μM, matching the value of the nonlabeled E255C/D428C SR1 variant but lower than the value of ∼3.5 μM for the WT, measured in bulk ATP hydrolysis experiments (Figure S2A,B). This observation can be explained considering that the SR1 construct measured in smFRET experiments consists of one labeled monomer and six WT monomers. Consequently, the apparent binding constant might be determined by the “local” properties of the observed labeled subunit. In contrast, the Hill coefficient—a quantity related to the strength of cooperativity between all the protomers in the complex—is governed by the global conformational behavior of the ring.

In line with the findings for the concerted allosteric model, we can treat the dynamic apo and ATP macrostates as representative signatures of the familiar T and R allosteric states of GroEL, respectively. By calculating the inter-microstate equilibrium constants of the two macrostates (readily obtained from the transition rates extracted from the single-molecule experiments), we can generate a closed thermodynamic cycle between the T and R states via the microstates, as shown in Figure 4A. This analysis allows us to estimate which microstate is likely to serve as the preferred gateway for the transition from T to R. It is assumed that transitions from T to R occur within each microstate, i.e., without crossing microstates. The equilibrium constants for the horizontal transitions between the microstates (in the direction from 1 to 4) are shown above and below the arrows, and the equilibrium constants for the vertical T to R transitions are calculated relative to the transition from microstate 1, labeled α_1_. In comparison to all four possible allosteric transitions, the scheme shows that the transition from conformational microstate 3, associated with the binding of ATP, is energetically the most favorable. When compared to the transition from microstate 2, the transition from microstate 3 yields an energetic gain of ∼0.8 kcal·mol^–1^ at the experimental temperature of ∼22 °C. While this contribution is modest, the thermodynamic cycle demonstrates the importance of subunit conformational dynamics, namely, that a fast exchange between the microstates grants the GroEL subunit more opportunities to reach the optimal microstate for a functional event, in this case, the allosteric transition.

**Figure 4:**
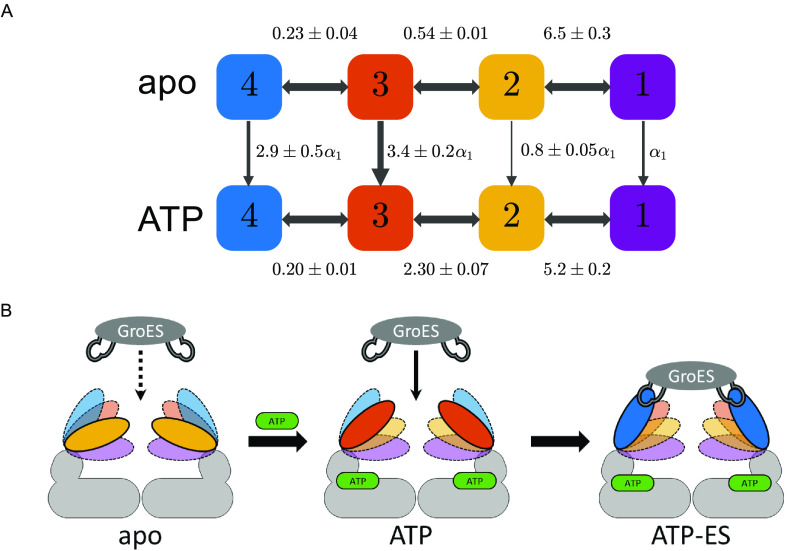
(A) Closed thermodynamic cycle between the two macrostates apo and ATP. The intermicrostate transitions are shown with horizontal arrows with the relevant equilibrium constants calculated as *K*_*i*__→i+1_ = *k*_*i*__+1_/*k*_*i*_, where *k*_*i*_, *k*_*i*__+1_ are the mean microstate transition rates reported in Figure 2A,B. The apo to ATP transitions are marked with vertical arrows together with the corresponding equilibrium constants, scaled relative to the transition from microstate 1 marked as α_1_ (also represented by arrow width). Mean values are shown with standard errors derived from repeats of the experiment. (B) Fast conformational motions and conformational selection. Illustrated is the GroEL ring with the equatorial and intermediate domains in gray and apical domain in other colors. The four microstates are represented by the different colors and positions of the apical domains. In each macrostate, the most occupied microstate is highlighted with a solid line. In the apo macrostate, the closed microstate 2 is mostly sampled, resulting in low affinity between the apical domains and the GroES mobile loops. In the ATP macrostate, microstate 2 is mostly sampled, together with a slight increase in the occupancy of microstate 1. As a result, the GroES mobile loops can bind with higher affinity to the apical domains, leading to the long-lived ATP-ES macrostate.

As has been shown in the past, fast conformational dynamics have a widespread, fundamental functional role in various biological processes, e.g., in promoting enzyme catalysis,^68,69^ molecular machine regulation,^70,71^ and protein disaggregation.^72,73^ Here we showed that GroEL also exhibits fast conformational motions, shuttling between four microstates, which are associated with various functional aspects in the chaperonin reaction cycle. What could be the significance of fast conformational motions at a submillisecond time scale in the case of GroEL? As a chaperone, GroEL interacts via its apical domains with various non-native protein substrates in a promiscuous manner since unfolded or misfolded proteins typically lack a specific structure.^7−10^ During the time window of the formation of a collision complex between GroEL and a protein substrate, fast domain motions can facilitate multiple binding and release opportunities between the apical domains and the exposed hydrophobic regions on the protein substrate, thus, promoting its unfolding reaction. It would be interesting to further investigate the dynamic behavior of GroEL in the presence of different protein substrates. Based on the findings on ref (38), a decrease in the transition rates between microstates is expected. This fast conformational motion of the GroEL subunit can also be related to the emergence of asymmetries among the ring subunits, which were observed in structural studies^34,35^ and simulations.^60^ As the thermodynamic cycle in Figure 4A implies, fast conformational motions may also be significant for the much slower functional cycle of the chaperone, as the recurrent population of all microstates facilitates, through conformational selection, the transition to the next step in the cycle, e.g., binding of ATP or the cochaperone GroES (Figure 4B).

To conclude, the intrinsic ability of the GroEL complex to frequently sample multiple conformational states and modulate its functional capabilities allows it to maintain a continuous capacity to respond to a variety of internal and external molecular events occurring on various time scales in the complex cellular environment. GroEL responds to external signals by changing the relative population of its submillisecond-exchanging conformational states. The single-molecule measurements monitoring changes in a single subunit of GroEL agree well with the results obtained by bulk measurements for an entire ring. This agreement provides new and strong evidence of the concerted allosteric transition of all seven subunits. We believe that fast conformational dynamics are a common and fundamental feature of many biomolecular processes that, with the advent of more sophisticated single-molecule measurement and analysis techniques, can now be readily observed and investigated.

## Supporting Information Available

Methods including protein purification, labeling and reassembly protocols, single-molecule data acquisition, and analysis, Supporting Figures S1–S11, and Supporting Tables S1–S3 (PDF)
